# Supplementary material for: D-Dimer Level and Neutrophils Count as Predictive and Prognostic Factors of Pulmonary Embolism in Severe Non-ICU COVID-19 Patients
Source: Viruses. 2021 Apr 26;13(5):758. doi: 10.3390/v13050758 (PMC8146364; doi:10.3390/v13050758)
Supplement: Supplementary file 1 [file viruses-13-00758-s001.zip › viruses-1186496-supplementary.pdf]

Table S1. Laboratory data upon admission and difference of paired biomarker data between the value at PE suspicion and the admission.

.	All *N*=86	PE *N*=30	NON-PE *N*=56	*P*-value
**Laboratory Parameters Upon Admission**				
Platelets [G/L]	222 [176-279]	222 [167-279]	223 [177-279]	0.8820
WBC [G/L]	7.5 [5.8-9.6]	9.4 [6.9-10.5]	6.8 [5.2-8.7]	**0.0088**
Lymphocytes [G/L]	0.9 [0.7-1.2]	0.9 [0.7-1.2]	0.9 [0.8-1.3]	0.2688
Neutrophils [G/L]	5.4 [4.0-8.4]	7.0 [4.7-8.9]	5.2 [3.9-6.7]	0.0706
C Reactive Protein, [mg/L]	108 [65-167]	112 [73-166]	108 [65-161]	0.7692
Ferritin [µg/L]	975 [479-1666]	1139 [626-2152]	968 [441-1356]	0.2212
BNP [pg/mL]	56 [11-198]	65 [30-407]	47 [10-195]	0.2873
Troponin [ng/L]	12 [4-22]	12 [5-19]	12 [4-27]	0.8817
Serum creatinine [µmol/L]	80 [61-116]	80 [60-120]	83 [63-112]	0.9003
Prothrombin time ratio [%]	88 [78-96]	83 [75-89]	90 [80-98]	**0.0446**
Fibrinogen [g/L]	6.4 [5.0-7.4]	6.0 [4.4-7.3]	6.4 [5.1-7.4]	0.4976
D-dimer [ng/mL]	2250 [1065-5025]	4335 [2432-17315]	1343 [763-2736]	**0.0008**
**Difference Of Paired Biomarker Data**				
Paired D-dimer difference (ng/mL), mean±SD	1988 ± 5381	3669 ± 7894	892 ± 2422	0.0584
Paired WBC difference (G/L), mean±SD	-0.2 ± 4.0	0.4 ± 5.2	-0.5 ± 3.1	0.4494
Paired Neutrophil count difference (G/L), mean±SD	-0.2 ± 4.0	0.2 ± 5.1	-0.4 ± 3.4	0.6452

Data were represented by median with IQR, and compared by Mann-Whitney U-test. For each paired variable, the value represents the mean ± SD difference calculated between the value at PE suspicion during hospitalization and upon admission. Values were compared in overall population with a paired Mann-Whitney U-test.

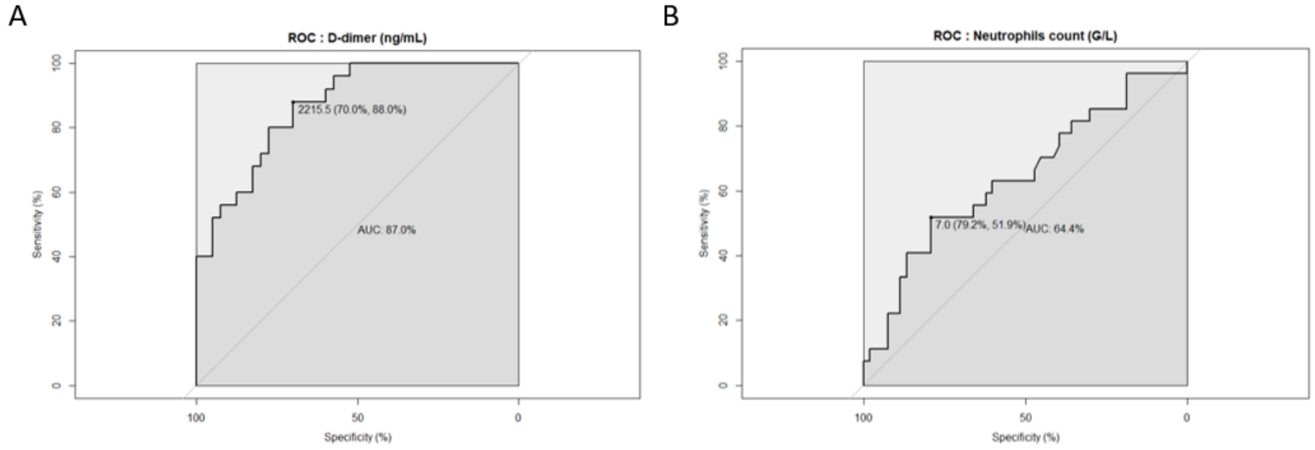

Figure S1. Receiver-operating characteristics (ROC) curve of biomarkers identified as predictive factors of pulmonary embolism during medicine ward hospitalization for non-ICU COVID-19. ROC curve and area under curve (AUC) of D-dimer is in the panel **A**, and of neutrophils count in the panel **B**. The threshold corresponded to the best sensitivity-specificity ratio, with in brackets the specificity displayed, followed by the sensitivity.

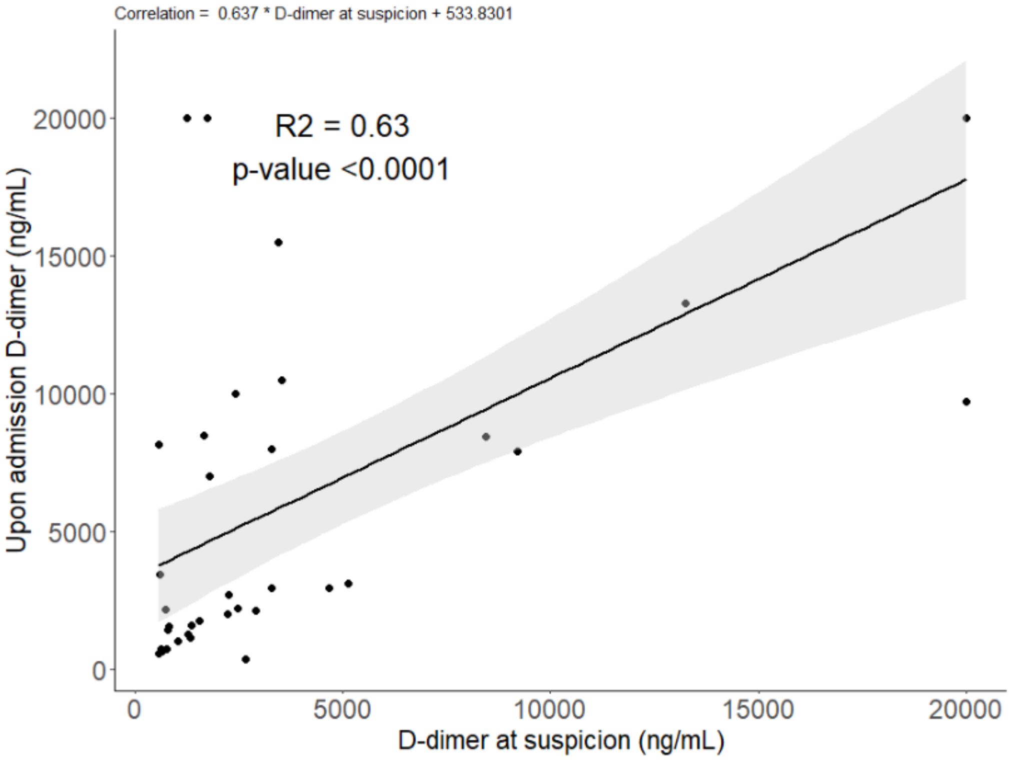

Figure S2. Correlation between D-dimer level upon admission and level at the suspicion time of PE evaluated by Pearson coefficient with a linear regression model.

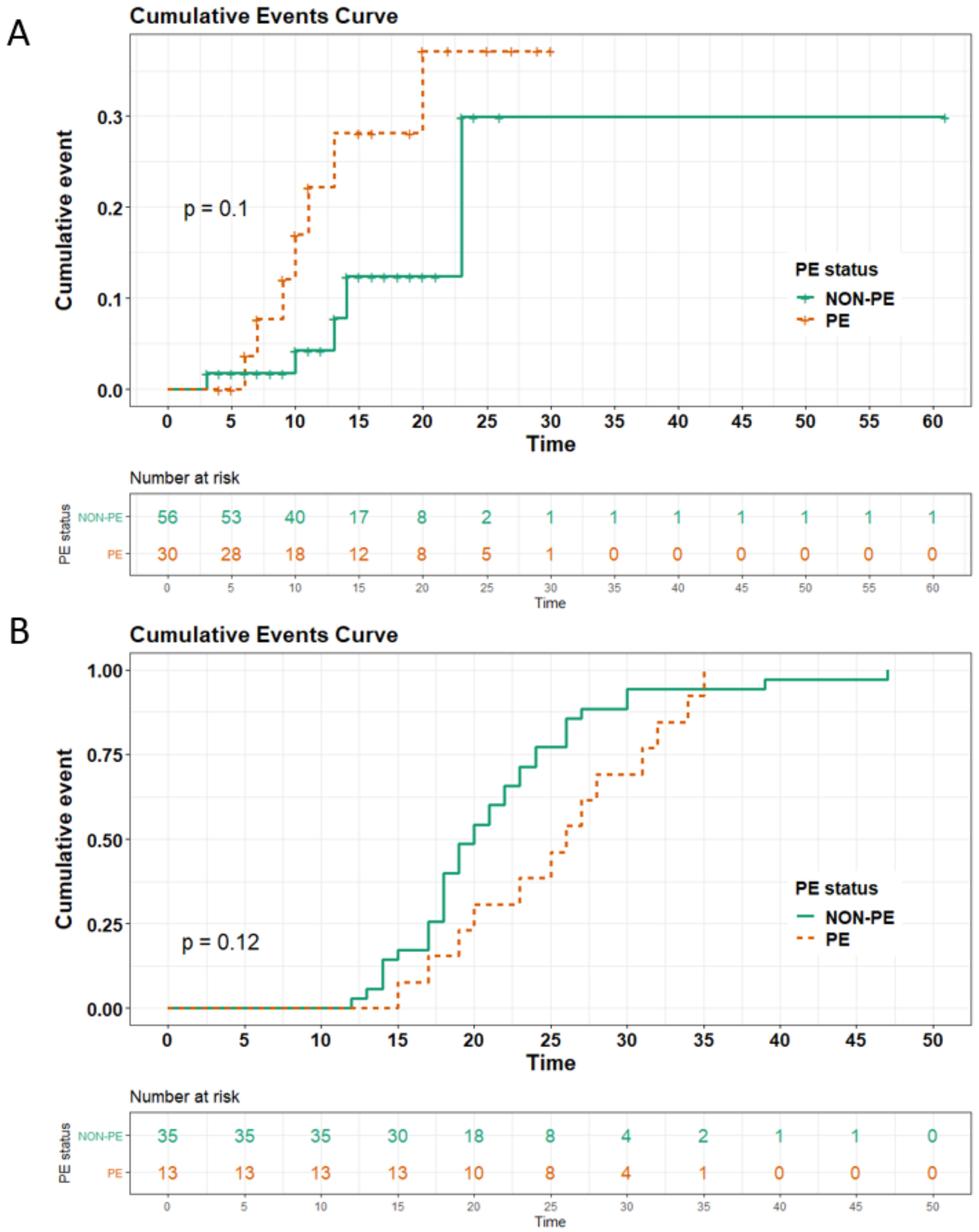

Figure S3. Cumulative events curve of ICU transfer or death in panel **A** and time to hospitalization discharge in panel **B** depending to the pulmonary embolism (PE) status. The "*p*" represents the global *p*-value of Logrank test in the Cox model. The risk table represents the patients still at risk of the event.
